# Association between *Paraoxonase* 1 polymorphisms and risk of esophagogastric junction adenocarcinoma: a case-control study involving 2,740 subjects

**DOI:** 10.18632/oncotarget.20104

**Published:** 2017-08-10

**Authors:** Weifeng Tang, Jianchao Liu, Yafeng Wang, Yanchao Chen, Mingqiang Kang, Jun Yin, Chao Liu, Jing Lin, Yu Chen

**Affiliations:** ^1^ Department of Cardiothoracic Surgery, Affiliated People's Hospital of Jiangsu University, Zhenjiang, Jiangsu Province, China; ^2^ Department of Cardiology, The People's Hospital of Xishuangbanna Dai Autonomous Prefecture, Jinghong, Yunnan Province, China; ^3^ Department of Thoracic Surgery, Affiliated Jurong People's Hospital of Jiangsu University, Jurong, Jiangsu Province, China; ^4^ Department of Thoracic Surgery, Fujian Medical University Union Hospital, Fuzhou, Fujian Province, China; ^5^ Key Laboratory of Ministry of Education for Gastrointestinal Cancer, Fujian Medical University, Fuzhou, Fujian Province, China; ^6^ Fujian Key Laboratory of Tumor Microbiology, Fujian Medical University, Fuzhou, Fujian Province, China; ^7^ Department of Medical Oncology, Fujian Cancer Hospital and Fujian Medical University Cancer Hospital, Fuzhou, Fujian Province, China; ^8^ Cancer Bio-immunotherapy Center, Fujian Cancer Hospital and Fujian Medical University Cancer Hospital, Fuzhou, Fujian Province, China; ^9^ Fujian Provincial Key Laboratory of Translational Cancer Medicine, Fuzhou, Fujian Province, China

**Keywords:** PON1, polymorphism, esophagogastric junction adenocarcinoma

## Abstract

Esophagogastric junction adenocarcinoma (EGJA) is a serious public health problem with high mortality in China. In this study, we assessed the association between *Paraoxonase 1* (*PON-1*) rs662 C>T, rs854560 A>T polymorphisms and EGJA risk. This case-control study enrolled 2,740 participants of Asians origin from the Eastern Chinese Han populations. SNPscanTM genotyping assay was harnessed to determine the genotyping of *PON1* polymorphisms. The *PON-1* rs854560 A>T and rs662 C>T genotypes distribution accorded with Hardy–Weinberg equilibrium. We found that there was no difference in the frequency of *PON-1* rs662 C>T, rs854560 A>T genotypes between the overall EGJA cases and controls. In the subgroup analyses, the results indicated that *PON-1* rs662 C>T polymorphism might be associated with a significantly decreased risk of EGJA in ever smoking group (TT vs. CC/CT: adjusted OR = 0.58, 95% CI 0.35–0.95, *P* = 0.029). In conclusion, our study highlights *PON-1* rs662 C>T polymorphism may decrease the risk of EGJA, which interacts with the tobacco using. In the future, a fine-mapping case-control study with detailed gene-environmental data is needed to further assess these potential relationship.

## INTRODUCTION

Esophagogastric junction adenocarcinoma (EGJA) is a common malignancy in North America, Europe and Eastern Asian and is considered that it is different from distal GC [1–3]. In the United States, the data of cancer registry program showed the incidence of EGJA increased 2.5-fold from 1973–1992, and it was stable in the last two decades with 2 per 100,000 morbidity [4]. In Caucasians, the 5-year relative survival rate was 16–17% [4, 5]. It is also reported that EGJA is a serious public health problem with high mortality in China. Liu *et al.* reported that the incidence of EGJA was 34.1% in all gastric and esophageal adenocarcinomas patients [6]. And 5-year survival rate of EGJA was 29–35.5% [7, 8]. The pathological process of EGJA is very complex. The etiology of EGJA is unclear. It is believed that food preserved by salting, chronic gastroesophageal reflux disease, smoking, obesity and etc. may contribute to the development of EGJA. Nowadays, accumulating evidences highlighted that individual's genetic background could play a vital role in the carcinogenesis of EGJA. Single nucleotide polymorphisms (SNPs), which could alter the activity of some detoxifying carcinogenic substances, may conduce to the development of EGJA.

Chronic inflammation may influence the susceptibility of malignancy. The potential molecular mechanisms underlying the relationship have been studied and it is identified that a number of inflammation-related cells gathers and secretes some chemical mediators, in particular reactive oxygen species (ROS) [9]. ROS levels within cells and inflammatory tissues are regulated by many free-radical scavenging systems. The excessive ROS damage intracellular macromolecules, including proteins and nucleic acids. *Paraoxonase 1* (*PON1*) gene maps to the long arm of chromosome 7. PON1, an antioxidant enzyme, keeps the balance of antioxidant–oxidant [10–13]. Enzyme Commission of the International Union of Biochemistry and Molecular Biology classifies PON1 as an aryldialkylphosphatase (EC 3.1.8.1) [14]. PON1, a glycoprotein, is a molecular mass of 43KDa. Oxidative stress have been found to be correlated with an increased susceptibility of many malignancies [9]. *In vivo*, body possesses several free-radical scavenging systems including paraoxonase. PON1 prevents the oxidation of LDL and cell membrane, and therefore it is thought to be atheroprotective. Furthermore, PON1 was found to play an important role in the scavenging of carcinogenic lipid-soluble radicals [15]. In addition, PON1 is versatile and it may contribute to innate immunity, putative new roles in malignancy and the promotion of healthy aging [16]. It was reported that expression or activity of PON1 decreased in lung cancer, multiple myeloma and papillary thyroid cancer [17–19]. Therefore, it is reasonable to believe that the decreased activity of PON1 may be associate with the development of cancer.

*PON1* rs662 C>T and rs854560 A>T, two functional SNPs, were identified to be associated with the risk of multiple cancers [20–22]. However, most of the epidemiologic and molecular studies focused on the relationship of *PON1* polymorphisms with the risk of cancer in Caucasians. Thus, the results might remain inconclusive, especially in Asians. Therefore, we conducted this case-control study to determine the association between *PON1* rs662 C>T, rs854560 A>T polymorphisms and EGJA risk with a large sample size in Eastern Chinese Han populations.

## RESULTS

### Baseline characteristics

The relevant demographics and risk factors are summarized by case/control status in Table 1. EGJA patients and non-cancer controls were similar in terms of age and sex distributions. There were more smokers and drinkers among EGJA patients than among controls. The minor allele frequency (MAF) of *PON-1* rs854560 A>T and rs662 C>T polymorphisms in controls was similar to its data in the database (Table 2). In controls, as demonstrated in Table 2, the *PON-1* rs854560 A>T and rs662 C>T genotypes distribution accorded with Hardy–Weinberg equilibrium (HWE).

**Table 1:** Distribution of selected demographic variables and risk factors

Variable	Overall Cases (*n* = 1,063)		Overall Controls (*n* = 1,677)		*P*^a^
	*n*	%	*n*	%	
Age (years)	64.19 (± 8.63)	63.91 (± 10.22)	0.451		
Age (years)					0.165
< 64	494	46.47	825	49.19	
≥ 64	569	53.53	852	50.81	
Sex					0.909
Female	304	28.60	483	28.80	
Male	759	71.40	1194	71.20	
Smoking status					< 0.001
Never	773	72.72	1323	78.89	
Ever	290	27.28	354	21.11	
Alcohol use					< 0.001
Never	908	85.42	1507	89.86	
Ever	155	14.58	170	10.14	

^a^ Two-sided *χ*^2^ test and Student t test.

**Table 2:** Primary information for *PON1* polymorphisms (rs854560 A>T and rs662 C>T)

Genotyped polymorphisms	rs854560 A>T	rs662 C>T
Chr	7	7
NCBI Build 38^a^	95316772	95308134
Function^a^	missense (dbSNP)	missense (dbSNP)
Minor allele frequency a for Chinese in database ^a^	0.03	0.43
Minor allele frequency in our controls (*n* = 1,677)	0.03	0.36
*P* value for HWE^b^ test in our controls	0.733	0.632
% Genotyping value	99.09	99.09

^a^ http://gvs.gs.washington.edu/GVS147/

^b^HWE: Hardy–Weinberg equilibrium.

### Association of *PON-1* rs662 C>T and rs854560 A>T polymorphisms with EGJA

The *PON-1* rs854560 A>T and rs662 C>T genotypes are summarized in Table 3. The frequencies of *PON-1* rs854560 AA, AT, and TT genotypes were 93.28%, 6.63% and 0.10% in EGJA group and 93.97%, 5.91%, and 0.12% in controls, respectively. When the frequency of *PON-1* rs854560 AA genotype was used as reference, there was no difference in the frequency of *PON-1* rs854560 AT genotype between the EGJA group and the controls (crude OR = 1.11, 95% CI: 0.81–1.52, *P* = 0.533). When the frequency of *PON-1* rs854560 AA genotype was used as reference, we found no difference in the frequency of *PON-1* rs854560 TT genotype between EGJA group and the controls (crude OR = 0.79, 95% CI: 0.07–8.76, *P* = 0.850). In addition, when the frequency of *PON-1* rs854560 AA genotype was used as reference, there was no difference in the frequency of *PON-1* rs854560 AT/TT genotypes between EGJA group and the controls (crude OR = 1.12, 95% CI: 0.82–1.54, *P* = 0.471). When the frequency of *PON-1* rs854560 AA/AT genotypes were used as reference, we found there was no difference in the frequency of *PON-1* rs854560 TT genotype between EGJA group and the controls (crude OR = 0.80, 95% CI: 0.07–8.88, *P* = 0.859). Adjustment for age, sex, smoking and drinking, the similar results were also found (AT vs. AA: adjusted OR, 1.12; 95% CI, 0.81–1.54; *P* = 0.488; TT vs. AA: adjusted OR, 0.90; 95% CI, 0.08–9.98; *P* = 0.933; TT/AT vs. AA: adjusted OR, 1.14; 95% CI, 0.83–1.56; *P* = 0.417; TT vs. AA/AT: adjusted OR, 0.92; 95% CI, 0.08–10.16; *P* = 0.945; Table 4).

**Table 3:** The frequencies of *PON1* rs854560 A>T and rs662 C>T polymorphisms

Genotype	Overall EGJA case (*n* = 1,063)		Overall Controls (*n* = 1,677)	
	*n*	%	*n*	%
rs854560 A>T				
AA	971	93.28	1573	93.97
AT	69	6.63	99	5.91
TT	1	0.10	2	0.12
AT + TT	70	6.72	101	6.03
AA + AT	1040	99.90	1,672	99.88
T allele	71	3.41	103	3.08
rs662 C>T				
CC	408	39.19	691	41.28
CT	501	48.13	776	46.36
TT	132	12.68	207	12.37
TT + CT	633	60.81	983	58.72
CT + CC	909	87.32	1,467	87.63
T allele	765	36.70	1,190	35.54

EGJA patients and controls.

**Table 4:** Analyses of the association between *PON1* rs854560 A>T, rs662 C>T polymorphisms and the risk of EGJA

Genotype	Overall (1,063 cases vs. 1,677 controls)			
	Crude OR (95%CI)	*P*	Adjusted ORa (95%CI)	*P*
rs854560 A>T				
additive model	1.11 (0.81–1.52)	0.533	1.12 (0.81–1.54)	0.488
homozygote model	0.79 (0.07–8.76)	0.850	0.90 (0.08–9.98)	0.933
Dominant model	1.12 (0.82–1.54)	0.471	1.14 (0.83–1.56)	0.417
Recessive model	0.80 (0.07–8.88)	0.859	0.92 (0.08–10.16)	0.945
rs662 C>T				
additive model	1.04 (0.88–1.23)	0.624	1.04 (0.88–1.23)	0.651
homozygote model	1.03 (0.80–1.32)	0.821	1.01 (0.79–1.30)	0.929
Dominant model	1.09 (0.93–1.28)	0.282	1.09 (0.93–1.27)	0.315
Recessive model	1.03 (0.82–1.30)	0.809	1.01 (0.80–1.28)	0.915

^a^Adjusted for age, sex, smoking status and alcohol use in a logistic regression model.

The frequencies of *PON-1* rs662 CC, CT, and TT genotypes were 39.19%, 48.13% and 12.68% in EGJA group and 41.28%, 46.36%, and 12.37% in controls, respectively. When the frequency of rs662 CC genotype was used as reference, there was no difference in the frequency of *PON-1* rs662 CT genotype between the EGJA group and the controls (crude OR = 1.04, 95% CI: 0.88–1.23, *P* = 0.624). When the frequency of *PON-1* rs662 CC genotype was used as reference, we found there was no difference in the frequency of *PON-1* rs662 TT genotype between EGJA group and the controls (crude OR = 1.03, 95% CI: 0.80–1.32, *P* = 0.821). In addition, when the frequency of *PON-1* rs662 CC genotype was used as reference, there was no difference in the frequency of *PON-1* rs662 CT/TT genotypes between EGJA group and the controls (crude OR = 1.09, 95% CI: 0.93–1.28, *P* = 0.282). When the frequency of *PON-1* rs662 CC/CT genotypes were used as reference, no difference was found in the frequency of *PON-1* rs662 TT genotype between EGJA group and the controls (crude OR = 1.03, 95% CI: 0.82–1.30, *P* = 0.809). Adjustment for age, sex, smoking and drinking, the similar results were also found (CT vs. CC: adjusted OR, 1.04; 95% CI, 0.88–1.23; *P* = 0.651; TT vs. CC: adjusted OR, 1.01; 95% CI, 0.79–1.30; *P* = 0.929; TT/CT vs. CC: adjusted OR, 1.09; 95% CI, 0.93–1.27; *P* = 0.315; TT vs. CC/CT: adjusted OR, 1.01; 95% CI, 0.80–1.28; *P* = 0.915; Table 4).

### Association of *PON-1* rs662 C>T and rs854560 A>T polymorphisms with EGJA in Different Stratification Groups

The *PON-1* rs854560 A>T genotype frequencies in the different stratified analyses are summarized in Table 5. We found that *PON-1* rs854560 A>T polymorphism was not associated with the risk of EGJA in any subgroup (Table 5).

**Table 5:** Stratified analyses between *PON-1* rs854560 A>T polymorphism and EGJA risk by sex, age, smoking status and alcohol consumption

Variable	*PON-1* rs854560 A>T (case/control) a			Adjusted OR b (95% CI); P				
	AA	AT	TT	AA	AT	TT	AT/TT	TT vs. (AA/AT)
Sex								
Male	696/1222	49/68	1/1	1.00	1.17 (0.80–1.71); *P*: 0.422	1.84 (0.12–29.48); *P*: 0.667	1.20 (0.82–1.75); *P*: 0.347	1.86 (0.12–29.79); *P*: 0.661
Female	275/451	20/31	0/1	1.00	0.95 (0.53–1.72); *P*: 0.875	-	0.96 (0.53–1.72); *P*: 0.885	-
Age								
< 64	449/777	32/45	1/1	1.00	1.19 (0.74–1.90); *P*: 0.479	2.00 (0.12–32.03); *P*: 0.625	1.23 (0.77–1.96); *P*: 0.383	2.03 (0.13–32.61); *P*: 0.617
≥ 64	522/796	37/54	0/1	1.00	1.03 (0.66–1.59); *P*: 0.908	-	1.03 (0.67–1.59); *P*: 0.894	-
Smoking status								
Never	700/1,239	53/80	1/2	1.00	1.13 (0.79–1.62); *P*: 0.501	0.89 (0.08–9.84); *P*: 0.923	1.16 (0.81–1.65); *P*: 0.429	0.90 (0.08–10.00); *P*: 0.934
Ever	271/334	16/19	0/0	1.00	1.04 (0.52–2.08); *P*: 0.919	-	1.05 (0.52–2.10); *P*: 0.900	-
Alcohol consumption								
Never	823/1414	63/89	1/2	1.00	1.18 (0.84–1.64); *P*: 0.342	0.89 (0.08–9.83); *P*: 0.923	1.20 (0.86–1.67); *P*: 0.285	0.90 (0.08–9.98); *P*: 0.932
Ever	148/159	6/10	0/0	1.00	0.58 (0.20–1.70); *P*: 0.320	-	0.58 (0.20–1.71); *P*: 0.325	-

^a^ The genotyping was successful in 1063 (97.93%) EGJA cases, and 1677 (99.82%) controls for *PON-1* rs854560 A>T.

^b^ Adjusted for age, sex, smoking status and alcohol consumption (besides stratified factors accordingly) in a logistic regression model.

The *PON-1* rs662 C>T genotype frequencies in the different stratified analyses are summarized in Table 6. After adjustment by logistic regression analysis, the results indicated that *PON-1* rs662 C>T polymorphism might be associated with a significantly decreased risk of EGJA in ever smoking group [TT vs. CC/CT: adjusted OR = 0.58, 95% CI 0.35–0.95, *P* = 0.029 (Table 6)]. In other subgroups, we did not find any association between *PON-1* rs662 C>T polymorphism and the risk of EGJA (Table 6).

**Table 6:** Stratified analyses between *PON-1* rs662 C>T polymorphism and EGJA risk by sex, age, smoking status and alcohol consumption

Variable	PON–1 rs662 C>T (case/control)^a^			Adjusted OR b (95% CI); *P*				
	CC	CT	TT	CC	CT	TT	CT/ TT	TT vs. (CT/CC)
Sex								
Male	285/488	370/553	91/150	1.00	1.10 (0.90–1.34); *P*: 0.344	0.98 (0.73–1.32); *P*: 0.902	1.12 (0.93–1.35); *P*: 0.250	0.95 (0.72–1.25); *P*: 0.706
Female	123/203	131/223	41/57	1.00	0.90 (0.66–1.23); *P*: 0.504	1.08 (0.68–1.72); *P*: 0.740	1.01 (0.75–1.36); *P*: 0.962	1.18 (0.76–1.83); *P*: 0.452
Age								
< 64	197/340	228/383	57/100	1.00	0.96 (0.76–1.23); *P*: 0.765	0.90 (0.62–1.31); *P*: 0.592	1.01 (0.80–1.27); *P*: 0.969	0.94 (0.67–1.34); *P*: 0.749
≥ 64	211/351	273/393	75/107	1.00	1.11 (0.89–1.40); *P*: 0.363	1.11 (0.79–1.56); *P*: 0.556	1.16 (0.93–1.45); *P*: 0.183	1.07 (0.78–1.47); *P*: 0.694
Smoking status								
Never	301/539	350/628	103/154	1.00	0.94 (0.78–1.14); *P*: 0.546	1.13 (0.85–1.50); *P*: 0.403	1.04 (0.86–1.25); *P*: 0.693	1.20 (0.92–1.57); *P*: 0.188
Ever	107/152	151/148	29/53	1.00	1.40 (0.99–1.96); *P*: 0.054	0.69 (0.41–1.16); *P*: 0.160	1.23 (0.89–1.70); *P*: 0.216	**0.58 (0.35–0.95); P: 0.029**
Alcohol consumption								
Never	354/627	422/700	111/178	1.00	1.01 (0.85–1.21); *P*: 0.897	1.04 (0.80–1.37); *P*: 0.760	1.08 (0.91–1.27); *P*: 0.398	1.06 (0.83–1.37); *P*: 0.634
Ever	54/64	79/76	21/29	1.00	1.21 (0.74–1.99); *P*: 0.443	0.91 (0.45–1.81); *P*: 0.778	1.13 (0.70–1.81); *P*: 0.615	0.81 (0.43–1.53); *P*: 0.512

^a^ The genotyping was successful in 1063 (97.93%) EGJA cases, and 1677 (99.82%) controls for *PON-1* rs662 C>T.

^b^ Adjusted for age, sex, smoking status and alcohol consumption (besides stratified factors accordingly) in a logistic regression model.

## DISCUSSION

EGJA is thought to be an independent malignancy entirety of upper digestive tract tumors [23]. It is reported that the incidence of EGJA is increasing worldwide [1–3, 24]. A number of previous studies indicated that dietary habits, lifestyle (e.g. smoking and drinking *et al.*), oxidative and carbonyl stresses, and estrogens might play important roles in carcinogenesis [25–30]. *In vivo*, there are many antioxidant enzyme which may prevent genotoxic damage. PON1, an antioxidant enzyme, may play a vital role in keeping the balance of antioxidant–oxidant balance [11, 31]. Several studies reported that expression or activity of PON1 decreased in several cancers [17–19]. Considering the potential role of PON1 in carcinogenesis, we selected two most common *PON1* polymorphisms (rs662 C>T and rs854560 A>T) and assessed their susceptibility to EGJA. In this study, we identified that *PON1* rs662 C>T polymorphism was associated with the decrease the risk of EGJA in ever smoking subgroup.

It was found that expression or activity of PON1 was lower in cancer patients than controls [17–19]. Delimaris *et al.* reported that oxidative stress might contribute to pathogenesis of cancer involving the proliferation and malignancy conversion [32]. Previous studies demonstrated that the variants of the *PON1* could affect the activity of PON1 protein. Thus, it was necessary to predict whether *PON1* polymorphisms might influence the development of EGJA. In this case-control study, we aimed to determine the relationship between *PON1* polymorphisms and EGJA risk. We found that *PON1* rs662 T allele might decrease the risk of EGJA in ever smoking subgroup, suggesting that *PON1* rs662 C>T polymorphism might act as a protective factor for EGJA. Kahraman *et al.* found tobacco exposure increased oxidative stress and decreased paraoxonase-1 [33]. The coding region *PON1* rs662 C>T polymorphism (R192Q) leads to an amino acid substitution and determines a substrate dependent effect on activity. Eom *et al.* reported that *PON1* rs662 TT/CT carriers had the lower urinary 8-hydroxydeoxyguanosine and thiobarbituric acid reactive substances levels compared with rs662 CC carriers in lung cancer patients and decreased the risk of lung cancer [34]. Recently, the association between the decreased risk of cancer and *PON1* rs662 C>T polymorphism was also found in Asians [35]. Our findings were similar to the results of these studies. However, there were only a few case-control studies with small sample size conducted in Asians. The evidence might be limited. In the future, more case-control studies focusing on the relationship of *PON1* rs662 C>T polymorphism with cancer risk should be performed to confirm these potential associations.

There are some limitations which may be interpreted. Firstly, although the number of the participants was relatively large, when the stratification analyses were carried out for the age, sex, smoking and drinking status, resulting in insufficient capacity which limited the power of this study. Secondly, the enrolled participants were from local hospitals, which might lead to the bias. Thirdly, there were only two missense SNPs in *PON1* gene included in this case-control study. In the future, a fine-mapping study should be performed to further determine the potential association between the functional SNPs in *PON1* gene and EGJA risk. Fourthly, only the information of drinking and smoking was collected as major risk factor. Other potential risk factors [e.g. gastroesophageal reflux disease, obesity, Helicobacter pylori infection status and dietary behavior (low intake of fruit and veggies, pickled food consumption, and drinking beverages at high temperatures etc.)] were not considered. Fifthly, biomarkers for oxidative stress were not measured in our study. Finally, for lack of raw data from other lifestyles, we did not further assess the relationships for the potential interactions of gene-gene or gene-environment factors.

In conclusion, our study highlights *PON1* rs662 C>T polymorphism may be correlated with the decreased risk of EGJA which interacted with the tobacco using. In the future, a fine-mapping case-control study are needed to further assess the potential relationship between *PON1* SNPs and EGJA risk.

## MATERIALS AND METHODS

### Subjects

This case-control study enrolled 2,740 participants of Asians origin from the Chinese Han population. Cases (*n* = 1,063) were newly diagnosed EGJA patients at Fujian Medical University Union Hospital and Fujian Medical University Cancer Hospital from January 2014 to May 2016, and at Affiliated People's Hospital of Jiangsu University from January 2008 to November 2016. EGJA patients were included consecutively. Two experienced pathologists confirmed the diagnosis of EGJA for all cases. The major selection criterion for EGJA cases were: (a) all EGJA cases were Siewert II subtype; (b) Patients were Eastern Chinese Han population, and (c) EGJA was confirmed via histopathological examinations. The major exclusion criteria for EGJA cases were: (1) had a history of personal autoimmune disease, (2) EGJA cases who received prior chemoradiotherapy and (3) had a history of another malignancy. At the same time, non-cancer controls were recruited from the Physical Examination Center of these local hospitals. The control subjects had no history of autoimmune disorder or personal malignancy, and were frequency matched to EGJA patients by sex and age. These subjects have been reported in our previous study [36].

Two trained personnels interviewed each participant. The demographic and lifestyle characteristics were obtained by using a questionnaire. Information on smoking, drinking, age and sex was collected for the present study. All participants were informed and signed written consent to allow their blood samples to be genetically tested. Approval was given by the ethical committees of Jiangsu University and Fujian Medical University, in accordance with the Declaration of Helsinki.

### DNA extraction and genotyping

DNA from the participants was extracted from (EDTA)-anticoagulated blood samples with Promega DNA Kit (Promega, Madison, USA). The obtained DNA sample was stored at −80°C. Genotyping was carried out by using SNPscan^™^ assay (Genesky Biotechologies Inc., Shanghai, China) to determine the genotypes of *PON-1* rs854560 A>T and rs662 C>T polymorphisms. For quality control, 110 DNA samples randomly selected from 2,740 specimens were reanalyzed. The genotypes of *PON-1* rs854560 A>T and rs662 C>T polymorphisms were confirmed by another laboratory technicians. As shown in Table 2, the success rate of *PON-1* genotyping was both more than 99%.

### Statistical analysis

The HWE in controls was tested by the internet-based *χ*^2^ test (http://ihg.gsf.de/cgi-bin/hw/hwa1.pl). The categorical variables (e.g. genotype distributions, age, sex, smoking status, and alcohol consumption) were compared by using Chi-square test (*χ*^2^). The continuous variable was compared by using Student's t-test. To test the hypothesis of relationship of *PON-1* genetic polymorphisms with EGJA risk, multivariate logistic regression analyses were used. The SAS 9.4 statistical package (SAS Institute Inc., Cary, NC, USA) was harnessed to calculate the results.
